# Averting catastrophic tuberculosis costs in an Indian state: integration of Ayushman Bharat Arogya Karnataka with National Tuberculosis Elimination Program

**DOI:** 10.1016/j.lansea.2023.100327

**Published:** 2023-11-23

**Authors:** Suresh G. Shastri, Pooja Sancheti, Sharath Burugina Nagaraja, Gagana G. Dayananda, Pujari K. Srinivas, Murugesh Jayaprakash, Shivashankara N. Ninge Gowda, Aditi Krishnamurthy, Balu P. Srinivasa, Anil Singarajipura, Randeep Devendiran

**Affiliations:** a AB-ArK Cell, Commissionerate of Health & Family Welfare Services, Bangalore, Karnataka, India; b Department of Community Medicine, ESIC Medical College and PGIMSR, Bengaluru, India; c Suvarna Arogya Suraksha Trust, Bangalore, Karnataka, India; d Independent Public Health Consultant, Bangalore, India; e Centre for Digital Health, Artificial Intelligence, Research and Training, Basaveshwara Medical College and Hospital, Chitradurga, India; f Directorate of Health & Family Welfare Services, Bangalore, Karnataka, India

**Keywords:** Tuberculosis, Universal health coverage, Ayushman Bharat-PMJAY-Arogya Karnataka, Karnataka's model, Health insurance, TB elimination, NTEP integration

## Abstract

The WHO's “End TB” initiative aims to reduce catastrophic expenses, incidence, and mortality by 90%, 80%, and 0%, respectively by 2030 and Government of India has committed to reaching these goals by 2025. Despite tremendous progress, tuberculosis (TB) remains one of the main public health issues. To limit TB transmission and expedite reduction in incidence, further measures are needed. These milestones and objectives remain aspirational until we achieve “Universal access” to high-quality TB diagnosis and treatment. The goals of the study include outlining the process of ‘Ayushman Bharat-Pradhan Mantri Jan Arogya Yojana-Arogya Karnataka’ (AB-PMJAY-ArK) integration with the National TB Elimination Program (NTEP) in Karnataka, the types of TB patients who used AB-PMJAY-ArK services, and calculating the cost per TB patient at primary, secondary, and tertiary healthcare facilities, both public and private, stratified by type of service. Increased coverage, elimination of treatment delays, early and free treatment, and prevention of missing patients are benefits of integrating NTEP with Ayushman Bharat-PMJAY.

## Introduction

Tuberculosis (TB) is a significant public health issue globally, leading to high morbidity and mortality. Even before the COVID-19 pandemic, TB caused more deaths than other infectious diseases, including HIV/AIDS.^1^ In India, despite the implementation of the National TB Elimination Program (NTEP), providing high-quality care to TB patients has been challenging due to limited access to services in the public sector. India has set ambitious targets to decrease TB incidence, mortality, and catastrophic costs through the WHO's “End TB” plan by 2030.^2^ However, achieving these goals requires optimising TB diagnostic, case holding and treatment services, despite increased resources and political commitment.^3^

India has the highest burden of TB, with over 2.7 million new cases and an estimated 440,000 deaths annually, disproportionately affecting marginalised population.^1^^,^^2^^,^^4^ TB incidence has been increasing globally since 2020, reversing two decades of annual decreases. In India, the reduction in TB cases from 2015 to 2021 was only halfway towards the “End TB” milestone.^1^^,^^5^

The TB prevalence for Karnataka is 30.4 million cases per year as per the National TB prevalence survey whereas prevalence across the States varied from 13.7 to 74.7 million cases per year. The total notified cases in Karnataka was 58.86% in 2021 and 80.4% in 2022 with a mortality rate of 6% in 2021 & 2022.^4^

To address the financial and economic barriers hindering access to healthcare services for many Non-Communicable Diseases (NCDs) including TB, the National Health Assurance Scheme ‘Ayushman Bharat-Pradhan Mantri Jan Arogya Yojana’ (AB-PMJAY) was launched by the Government of India. The scheme provides Universal Health Coverage (UHC) by engaging both public and private sectors and covers a wide range of medical packages, including TB treatment procedures. The AB-PMJAY scheme, which encompasses promotive, preventive, palliative, and rehabilitative aspects of UHC, is integrated with the Arogya Karnataka scheme (ArK)^6^ and implemented with the State Health Agency-Suvarna Arogya Suraksha Trust (SAST). At present, based on the census data from 2011, the program caters to a total of 61.1 million beneficiaries. Out of this number, 50.9 million beneficiaries align with the targeted population as indicated by the Food Civil Supply data, while the remaining beneficiaries are covered by alternative insurance programs.

The aim of achieving UHC by improving access to TB diagnostic services at primary care level and eliminating financial barriers in accessing care, can be achieved by linking NTEP with ‘Ayushman Bharat-Pradhan Mantri Jan Arogya Yojana-Arogya Karnataka’ (AB-PMJAY-ArK) at the state and national level. This will bridge the case finding gap, reduce treatment delays, prevent TB-related deaths, eliminate catastrophic costs, and assist in achieving the “End TB” strategy. The study objectives include describing the levels and process of AB-PMJAY-ArK integration with the National TB Elimination Programme in Karnataka, describing the type of TB patients who utilised AB-ArK services at health facilities, and determining the cost incurred by the AB-PMJAY-ArK per TB patient at primary, secondary, and tertiary healthcare facilities, both public and private, stratified by type of service.

## Methods

The AB-PMJAY-ArK program offers 1650 inpatient treatment packages for various disorders, aiming to reduce out-of-pocket expenses and improve universal health coverage. The scheme is integrated with the entire healthcare system from primary healthcare centres to higher facilities, with every government facility and many private hospitals empanelled under the scheme. Patients can avail treatment across the country with national portability. The program is implemented by Suvarna Arogya Suraksha Trust in Karnataka, which works in a trust-based manner and promotes the expansion of government hospitals' service areas. SAST releases 50–75% of the treatment cost to all the government hospitals and 100% to empanelled private facilities.

In Karnataka, the public health sector is strengthened by two distinctive characteristics that enable it to offer more specialised services and expand in line with State and federal policies. First, there is the “ring fencing” of basic secondary procedures (2A), which limits the performance of 294 of the 1650 procedures to Public Health Institutes (PHIs) only. The second is the gatekeeping mechanism, which requires all 2B (complex secondary) and 3A (tertiary) procedures to be treated in government hospitals and may only be referred to empanelled private hospitals with the proper reference if the procedure is unavailable on a certain day in government hospitals. Our data shows how these initiatives have increased the public health sector's ability to combat TB and its complications and deliver TB services to every individual with financial support.

The SAST portal captures data of every patient availing treatment under the scheme, and a preauthorisation is raised once the patient is admitted with a unique AB-ArK reference identification number. We utilised aggregated Karnataka State's AB-PMJAY-ArK preauthorisation and claims data from the SAST portal for analysis. The period considered for analysis was 24 months from January 2021 to December 2022. We identified TB-related treatment packages from the 1650 treatment packages listed by SAST at the inception of the scheme.

Only confirmed TB cases were included in the study and presumptive or indicative TB and TB related cases/codes were excluded as AB-PMJAY-ArK scheme has 43 treatment packages for TB related cases (Table 1).

**Table 1:** List of TB specific treatment packages offered under the AB-ArK scheme.

Sl.No.	Speciality	Type	Procedure code	Procedure name
1	General Medicine	2A	2A.M1.00027	Pericardial or Pleural tuberculosis–routine ward
2	Neonatal and Paediatrics	2B	2B.M2.00050	Intracranial ring enhancing lesion with complication (neurocysticercosis, tuberculoma)–routine ward
3	Orthopaedics	2B	2B.S5.17,021	Anterolateral Clearance for Tuberculosis
4	Cardiothoracic Surgery	3A	3A.S13.00067	Encysted Empyema/Pleural Effusion–Tubercular
5	Neonatal and Paediatrics	4A	4A.M2.00035	Tuberculosis–routine ward

## Results

During the period between January 2021 and December 2022, a total of 3,243,290 pre-authorisations were considered for analysis, among which 7450 (0.2%) individuals were included who had confirmed TB disease.

The analysis of the study involved 7450 individuals who were diagnosed with TB, out of which 5024 (67.4%) were male and 2426 (32.6%) were female. Of these individuals, 76 (1%) were aged 0–5 years, 403 (5.4%) were aged 6–18 years, 819 (14.5%) were aged 19–30 years, 2101 (28.2%) were aged 31–45 years, 2222 (29.8%) were aged 46–60 years, and 1829 (24.6%) were aged 60 years or older. Moreover, 7230 (97%) belonged to the Below Poverty Line (BPL) category, while 220 (3%) belonged to the Above Poverty Line (APL) category (Table 2).

**Table 2:** Sociodemographic profile of diagnosed TB patients who availed the services under AB-ArK scheme, 2021 and 2022.

Characteristics	Frequency (%)
Gender	
Female	2426 (32.6)
Male	5024 (67.4)
Age	
0–5 years	76 (1)
6–18 years	403 (5.4)
19–30 years	819 (14.5)
31–45 years	2101 (28.2)
46–60 years	2222 (29.8)
>60 years	1829 (24.6)
Family type	
Below Poverty Line (BPL)	7230 (97)
Above Poverty Line (APL)	220 (3)
Type of TB	
Pulmonary TB	3071 (41.2)
Extra-Pulmonary TB	4379 (58.8)

Most individuals (95.3%) sought treatment from the government sector, while a smaller percentage (4.7%) sought treatment from the private sector. Among those who received treatment from the government sector, 96.9% belonged to the BPL category, and the remaining 3.1% belonged to the APL category. The government sector expenditures were mainly attributed to these individuals (89%). On the other hand, of those who sought treatment from the private sector, 98.5% belonged to the BPL category, while only 1.5% belonged to the APL category. The expenses for treatment from the private sector were largely attributed to these individuals (11%).

In terms of the type of healthcare facility, most patients (84.5%) were treated at government tertiary facilities, which also accounted for the highest expenditure of US$ 7.8 million. Private tertiary facilities, on the other hand, had the lowest number of patients (4.6%).

Our study found that the commonest occurrence of TB was observed in the pericardial or pleural site, accounting for 53.4% of the cases. The pulmonary site followed with 41.2%, while the spine was the least affected site, accounting for only 0.1% of the cases.

Of the patients admitted for TB treatment packages, 74% were admitted to routine wards, accounting for 62% of the total expenditure (ranging from US$ 54.54 – US$ 1012 per patient), while only 1.6% required ventilators, accounting for 4.3% of the total expenditure (ranging from US$ 211.51—US$ 1658.18 per patient). The Department of General Medicine managed about 71.6% of confirmed TB patients under AB-ArK, accounting for 71.5% of the expenditure, with Department of Pulmonology managing 24.1% of patients and accounting for 23.1% of the expenditure. The remaining patients were mostly managed by the Department of Paediatrics (Table 3).

**Table 3:** Utilisation of different specialities by TB patients, the amount incurred and the average cost per speciality AB-ArK scheme, 2021 & 2022.

Type of speciality	Number of TB patients (%)	Amount spent (US$)	Average cost per TB patient (US$)
General medicine	5331 (71.6%)	6,63,683.60	124.5
Pulmonology	1794 (24.1%)	2,14,375.40	119.5
Paediatric surgeries	308 (4.1%)	46,400.60	150.7
Cardiothoracic surgery	12 (0.2%)	2484.80	207.1
Orthopaedics	5 (0.1%)	818.2	163.6
**Grand total**	**7450**	**9,27,762.60**	**124.5**

The total pre-authorisation requests submitted during the period accounted for 3.2 million cases, with confirmed TB cases comprising only 0.22% (7450) in volume but amounting to US$ 9.27 million of the total financial out go of the scheme. The number of confirmed TB cases in financial year (FY) 2022–23 (April 2022–December 2022) doubled compared to FY 2021–22 (April 2021–March 2022) in just nine months, indicating a reporting lag due to COVID, while the average approved sum over the years has remained relatively stable, suggesting no significant increase in treatment costs. Of the patients treated, 96.9% were discharged, 1.4% left against medical advice, 0.5% were referred, and 1% died, with males accounting for 71% of the total deaths and females 29%. Regarding catastrophic costs incurred due to TB, 89.15% of confirmed TB cases were treated under the government sector, while only 10.85% were treated under private institutions, indicating the strength of the public health system in the State and its coverage of all possible TB treatments.

## Discussion

Our analysis indicates that convergence has had a positive impact on patient management for tuberculosis at PHIs by covering the out-of-pocket expenditure (OOPE) costs, resulting in increased hospitalisation rates in the public sector and better TB outcomes. Previous studies have also found higher hospitalisation rates in public health facilities compared to private facilities for TB.^7^

Hospitalisation expenses are a major component of the direct costs of treating TB, and inpatient treatment is often necessary. This significantly increases the risk of catastrophic expenses, especially in the private sector. In their work on cost analysis for TB treatment, P Sinha et al. recommended significant reimbursement for inpatient TB therapy.^8^

TB is a disease that can affect anyone regardless of age or gender. According to the National TB survey, adult men account for 56.5% of all TB cases in 2021, while adult women account for 32.5%. In our study, men accounted for a slightly higher percentage at 67.4%, while women accounted for 32.6%. Men are more likely than women to contract the disease, and gaps in case detection and reporting are more prevalent among men.^4^^,^^9^

A study by Bhargava and colleagues found that 64% of hospitalisations for TB occurred in the public sector, while 36% occurred in the private sector.^10^ In contrast, our study found that only 4% of TB patients were admitted to private hospitals due to the state's unique gatekeeping mechanism, which requires all simple secondary procedures to be performed in PHIs and complex secondary and tertiary cases to be referred to private hospitals only if treatment is unavailable based on capability modules.

According to a nationwide study by Kastor et al. on catastrophic cost and distress health financing, TB patients who need hospitalisation incur a minimum cost of US$ 159 without health insurance while our integrated scheme has provided a minimum coverage of US$ 124.5 per patient, thereby largely preventing health financing distress and OOPE.^7^ On an average, the AB-PMJAY-ArK has helped reduce the catastrophic cost of TB treatment by US$ 124.5 per patient, while the mean expenditure on hospitalisation for TB patients in the study by Bhargava and colleagues was US$ 159.^11^ Additionally, a study found that the fatality rate due to TB dropped from 29% to 4%, and the treatment effectiveness rate tripled from 25% to 86%.^12^ Furthermore, males had a higher mortality rate than females at all ages, as demonstrated in our study, with males accounting for 71% of TB-related deaths and females accounting for 29%.^9^

The incidence rate of TB per capita is significantly correlated with development indices such as average income and undernourishment. The high cost of hospitalisation in India acts as a barrier to accessing inpatient care for underprivileged TB patients, with 42% of TB-affected households resorting to distress financing such as taking out loans or selling assets.^7^^,^^11^ By providing comprehensive TB treatment plans, including tests and improved access to care, AB-PMJAY-ArK has significantly reduced distress financing and increased hospitalisation rates.

Early risk assessment and comprehensive clinical care, including access to high-quality inpatient services, can save more lives.^10^ However, in India, only a small percentage of TB-related deaths occur in hospitals, with an estimated 85% taking place at home due to institutional, economic, and systemic barriers.^13^ Therefore, AB-PMJAY-ArK has improved access to TB diagnostic services at the primary care level and secondary and tertiary care facilities through various strategies to remove cost barriers, reduce delays, and prevent TB-related deaths.

The National flagship health program, AB-PMJAY, is implemented throughout the country and offers 1350 treatment packages, including those for TB. The National Health Authority (NHA) allows for State-specific modifications. By integrating the relevant beneficiary database, the program can be extended to the entire Indian population. Many States have appointed Pradhan Mantri-Arogya Mitras (PM-AMs) in hospitals to facilitate coordination between beneficiaries and hospital staff under the AB-PMJAY scheme. As a result, TB cases reported under the NTEP can be brought to the attention of PM-AMs, who can verify eligibility and provide the necessary assistance. Eligible beneficiaries can be treated under AB-PMJAY, while others can be treated through NTEP, preventing any duplications and ensuring improved financial outcomes.

Furthermore, integration will expand access to the target audience and enhance coverage, as NTEP primarily relies on the Ni-kshay portal (online patient management system designed to support TB control). AB-PMJAY, being a nationwide program with active participation from every State, provides additional benefits as a UHC initiative, covering the entire population and reducing missed TB cases. This improves TB notification, as only 71% of TB cases in India were reported as of the 2022 TB report.^14^ According to the WHO Global TB report, a multisectoral approach is necessary to achieve global TB targets, including timely access to TB treatment and diagnosis without financial difficulties. Therefore, integrating such a scheme nationwide can significantly contribute to TB elimination and the achievement of Sustainable Development Goals (SDGs).

The WHO identifies service coverage and the percentage of the population facing catastrophic costs as two elements crucial for achieving UHC. In India, 19% of TB patients face catastrophic health costs. Thus, integration will play a role in reaching UHC in TB care.^15^ The synergy between the national AB-PMJAY and NTEP will not only increase coverage but also lead to improved health outcomes, timely services, easy access, efficient resource utilisation, and avoidance of duplication.

While AB-PMJAY-ArK's initiatives have strengthened the health system, certain strategies can be considered. Extending Inpatient (IPD) coverage within the program's umbrella is critical for achieving better TB outcomes. Additional travel costs, wage loss, and indirect OOPE must all be addressed. To achieve the End TB goal, the inclusion of nursing care as well as expanding follow-up care coverage must be prioritised to maintain the continuum of care cycle.

Furthermore, IT integration of Ni-kshay portal and SAST TMS (the State Health Agency portal) with ABHA (Ayushman Bharat Health Account) Identification number as the common identity as ABHA is a universal health identity for an Indian citizen which is already linked individually with AB-PMJAY-ArK as well as Ni-kshay portal and can improve coverage, avoid duplication and provide accessible care. To combat tuberculosis and improve health outcomes in terms of mortality and morbidity, we recommend inclusion of identification of severe cases and provide post-hospitalisation care, post TB sequalae or complication care and follow up.

## Conclusion

Integration of the National and State scheme as AB-PMJAY-ArK and linking the NTEP with AB-PMJAY-ArK has shown promising results leading to increased coverage, reducing OOPE, reduced treatment delays, early and free treatment and prevents missing patients. Overall, it provides comprehensive patient evaluations and clinical care.

## Contributors

Conceptualisation: SGS, PS, SBN, AK, BPS, RD. Data curation: PS and MJ. Formal analysis: SGS, PS, MJ, AK. Investigation: SGS, PS, GGD, MJ. Methodology: SGS, PS, SBN, GGD, PKS, MJ, AK, BPS, AS. Project administration: SGS, PS, PKS. Resources: PS, SBN, PKS, MJ, AK, BPS. Supervision: SGS, PKS, SN, RD. Validation: SGS, PS, SBN, PKS, MJ, AK. Visualisation: SGS, PS, SBN. Writing–original draft: SGS and PS. Writing–review & editing: SGS, PS, SBN, PKS, SN, AK, BPS, AS and RD.

## Data sharing statement

The data for the study has been taken from the Suvarna Arogya Suraksha Trust platform with due permission. The data will be made available on request to the corresponding author.

## Declaration of interests

The authors have no competing interests to declare. This study did not receive any specific grant from funding agencies in the public, commercial, or not-for-profit sectors.
